# Comparison of Mortality between Japanese Peritoneal Dialysis and Hemodialysis Patients: A 5-Year Multicenter Follow-Up Study

**DOI:** 10.1155/2012/231018

**Published:** 2012-05-30

**Authors:** Kazuko Suzuki, Tsuneo Konta, Kazunobu Ichikawa, Ami Ikeda, Hiroki Niino, Masato Hoshikawa, Toshiyuki Takahashi, Hiroshi Abiko, Minoru Ito, Ikuto Masakane, Tomohito Matsunaga, Kosuke Kudo, Hiroko Sato, Noriyuki Degawa, Isao Kubota

**Affiliations:** ^1^Department of Cardiology, Pulmonology, and Nephrology, Yamagata University School of Medicine, 2-2-2, Iida-Nishi, Yamagata, Yamagata 990-9585, Japan; ^2^Department of Internal Medicine, Okitama Public General Hospital, Kawanishi 992-0601, Japan; ^3^Department of Internal Medicine, Nihonkai General Hospital, Sakata 998-8501, Japan; ^4^Dialysis Center, Yabuki Hospital, Yamagata 990-0043, Japan; ^5^Dialysis Center, Keijinkai Hospital, Osaki 989-6117, Japan; ^6^Department of Internal Medicine, Yamagata City Hospital Saiseikan, Yamagata 990-0042, Japan

## Abstract

To examine the relationship between dialysis modality and prognosis in Japanese patients, we conducted a prospective multicenter observational study. We recruited 83 background-matched peritoneal dialysis (PD) and 83 hemodialysis (HD) patients (average age, 64.9 years; men, 53.6%; diabetic patients, 22.9%; median duration of dialysis, 48 months in all patients) and followed them for 5 years. During the follow-up period, 27 PD patients (16 cardiovascular and 11 non-cardiovascular deaths) and 27 HD patients died (14 cardiovascular and 13 non-cardiovascular deaths). There were 8 PD patients switched to HD, and 6 PD patients received renal transplantation. Kaplan-Meier analysis revealed that the crude survival rate was not significantly different at the end of 5 years (PD 67.5% versus 67.5%, log-rank *P* = 0.719). The difference in cardiovascular and non-cardiovascular mortalities between PD and HD was not statistically significant. Multivariate Cox analysis showed that the independent predictors for death were age and serum albumin levels, but not the dialysis modality. This study showed that the overall mortality was not significantly different between PD and HD patients, which suggests that dialysis modality might not be an independent factor for survival in Japanese patients.

## 1. Introduction

The number of end-stage renal disease patients requiring renal replacement therapy is increasing worldwide. In Japan peritoneal dialysis (PD) and hemodialysis (HD) are the two major dialysis modalities. The prognosis associated with these modalities is a clinically important issue and has been debated for a long time.

Some studies show that PD is associated with a higher mortality than HD [1, 2]. Other studies show that PD patients have a higher survival rate than HD patients [3, 4]. Comprehensive analysis, including the results of large-scale and prospective studies, revealed that the HD and PD patients have a similar overall survival [5–7]. Another report indicated that the survival rates of PD and HD patients varied greatly depending on the characteristics of patients and observational period [8].

In Japan, only a few studies have been performed on this issue. A single-center study showed that the 3-year survival of PD patients was higher than that of the background-unmatched HD patients [9]. To clarify the difference in mortality between PD and HD patients, we performed a multicenter and background-matched analysis in Japanese dialysis patients.

## 2. Subjects and Methods

### 2.1. Patients

In our prospective cohort study, we recruited clinical parameter-matched 83 PD and 83 HD patients on maintenance dialysis from 6 hospitals in Yamagata and Miyagi prefectures, administrative districts with a population of 1.2 and 2.3 million, respectively, located in the northern part of Japan. The main purpose of this study was to analyze the factors related to survival in dialysis patients. Patients were registered in 2003 and were followed up until the end of 2008. The median follow-up period was 60 months. Baseline information was collected at entry, and the information for the prognosis was collected at the end of each year. Cardiovascular death was defined as the death due to coronary heart disease, heart failure and stroke, or cardiac sudden death that occurred within 1 h after the onset of acute symptoms. Patients recruited in the study gave written informed consent. This study was performed according to the Declaration of Helsinki and was approved by the institutional ethics committee.

### 2.2. Statistical Analysis

The means and proportions between groups were compared using unpaired Student *t*-test and chi-square tests, respectively. The Mann-Whitney *U*-test was used to compare the non-parametric data. Survival curves were estimated using the Kaplan-Meier method followed by the log-rank test. To examine the independent effect of the clinical parameters on prognosis, Cox-proportional hazard analysis was used with adjustment for potential confounders. The patients that changed the dialysis modality were censored at the time of changing the modality. Data are expressed as mean ± SD or median (min-max) for data not normally distributed. All statistical analyses were performed using Stat View version 5 (SAS Institute Inc., Cary, NC, USA). A significant difference was defined as *P* < 0.05.

## 3. Results

### 3.1. Patient Characteristics and Follow-Up

The baseline characteristics of the 166 patients, including 83 PD and 83 HD patients, were as follows: average age, 64.9 years; men, 53.6% (*n* = 89); diabetic patients, 22.9% (*n* = 38); median duration of dialysis, 48 months. The baseline clinical parameters including, age, gender, duration of dialysis, and the types of administered drugs were not significantly different between the PD and HD patients (Table 1).

During the 5-year follow-up period, there were 27 deaths in the PD group (16 cardiovascular and 11 noncardiovascular deaths), including deaths from congestive heart failure (*n* = 8), stroke (*n* = 5), and infection (*n* = 2). In HD group, there were 27 deaths (14 cardiovascular and 13 noncardiovascular deaths), including deaths from congestive heart failure (*n* = 6), ischemic heart disease (*n* = 3), and infection (*n* = 4). There were 8 PD patients switched to HD, and 6 PD patients received renal transplantation.

### 3.2. Association between Mortality and Dialysis Modality

**Figure 1:**
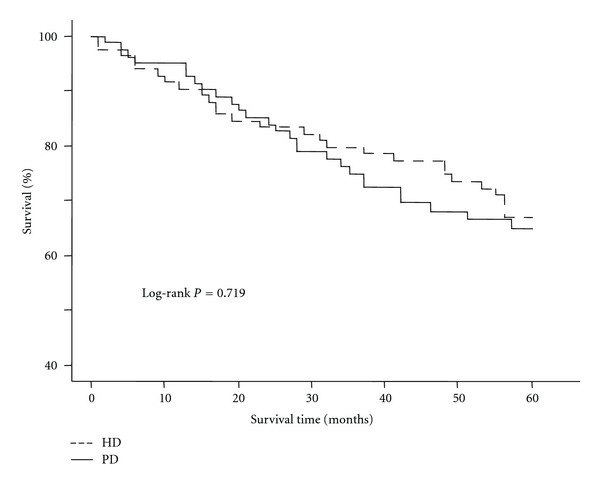
Kaplan-Meier survival curves by dialysis modality. PD: peritoneal dialysis; HD: hemodialysis.

The 5-year mortality associated with PD and HD was compared using Kaplan-Meier analysis. The survival rate was not significantly different at the end of 5 years (PD 67.5% versus 67.5%, log-rank *P* = 0.719) (Figure 1).

**Figure 2:**
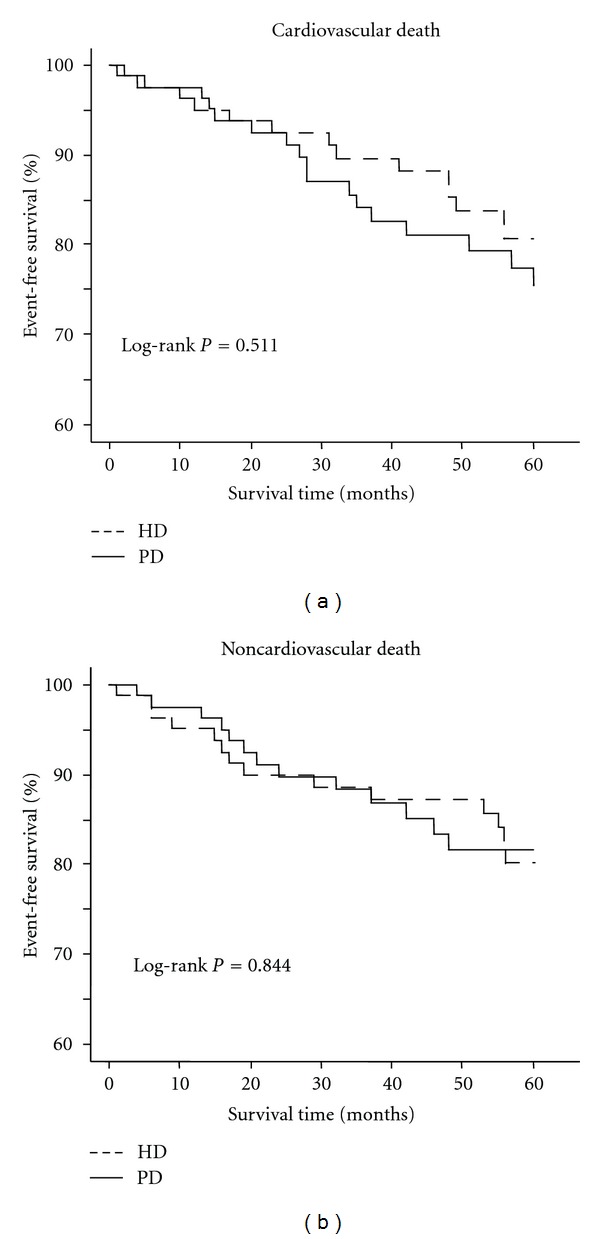
Cardiovascular and noncardiovascular mortalities by dialysis modality. PD: peritoneal dialysis; HD: hemodialysis.

Then, we examined the difference in cardiovascular and noncardiovascular mortalities between PD and HD patients. The proportion of cardiovascular deaths among total deaths was not significantly different between PD and HD (59.3% versus 51.2%, *P* = 0.584). The event-free curves of PD and HD patients were almost identical, and event-free rate at the end of 5 years was not significantly different in cardiovascular deaths (log-rank *P* = 0.511) and noncardiovascular deaths (log-rank *P* = 0.844) (Figure 2).

Next, we performed a Cox-proportional analysis to examine the independent effect of the dialysis modality on survival (Table 2). The univariate model showed that the factors associated with 5-year survival were age, past history of cardiovascular disease, serum albumin level, and triglycerides level but not the modality of dialysis. Multivariate analysis, including the factors significant according to univariate analysis and dialysis modality, showed that the independent predictors of survival were age (per 10 years increase, hazard ratio [HR], 1.89; 95% confidence interval [CI], 1.34–2.64; *P* < 0.001) and serum albumin level (per 1SD increase, HR, 0.67; 95% CI, 0.49–0.91; *P* = 0.011), and the modality of dialysis was not significantly associated with the outcome. Similarly, the modality of dialysis was not associated with the cardiovascular and noncardiovascular mortalities.

## 4. Discussion

In this prospective multicenter study, we compared the 5-year survival between background-matched HD and PD patients. Our results showed no significant difference in all-cause, cardiovascular, and noncardiovascular mortalities between HD and PD patients, which indicated that the dialysis modality might not be an independent factor for the survival in Japanese patients.

While many studies have compared the prognosis between PD and HD, consistent results remain to be obtained. One of the possible explanations is the difference in the observational period. Although studies with observational periods within 3 years are likely to detect a difference in survival between PD and HD patients [1, 3, 9], all the studies with long observational periods (longer than 10 years) showed no difference in prognosis [6, 7, 10].

Another possibility is the difference in the characteristics of participants. In Japan, PD is selected as renal replacement therapy in less than 5% of incident patients, which is much lower than that in other countries (10%–30% in most of European and Asian countries, and 8% in the United States). This suggests that there might be a bias in selecting the dialysis modality in Japan. The selection bias could be a confounding factor, and a proper adjustment for the background of subjects is indispensable for comparative analysis. Although a previous Japanese report showed that the survival was better in PD patients than in HD patients, the analysis was performed without adjusting for the background of the patients [9]. We performed an adjustment for the clinical parameters, and our results showed that the difference in survival rate was not significant at the end of 5 years. The discrepancy between our findings and those reported previously may be because of the difference in analytical methods, including the adjustment of background and the follow-up period.

Previous studies have shown that age, comorbidities, and nutritional parameters such as serum albumin level [11] and/or total cholesterol levels are the factors related to the prognosis in dialysis patients [12]. In the current study, serum albumin level was an independent predictor for 5-year survival. Our result is consistent with the previous finding and suggests that the nutritional status is more important than the dialysis modality in Japanese patients. Previously, infection was the leading cause of death in PD patients. However, the incidence of infection was decreased along with recent advances in connecting devices.

 Our study has advantages such as background-adjustment and relatively long observational period; however, there are several limitations to our study. First, we recruited patients undergoing dialysis not incident patients. Therefore, the dropout during the initial period of dialysis is not examined in this study. Second, a daily variation is observed in the clinical parameters. One time measurement of these parameters might underestimate the association between the parameters and the survival.

## 5. Conclusion

This background-matched 5-year observational study showed that all-cause, cardiovascular, and noncardiovascular mortalities were not significantly different between PD and HD patients in Japan.

## Figures and Tables

**Table 1:** Baseline characteristics of patients.

Characteristics	Peritoneal dialysis	Hemodialysis	* P* value
Number	83	83	
Mean age (years)	63.9 ± 12.7	65.9 ± 11.1	0.282
Men (%)	42.2	50.6	0.276
Duration of dialysis (months)	42 (5–310)	51 (9–305)	0.177
Body weight (kg)	55.5 ± 6.1	52.5 ± 7.4	0.066
Obesity (%)	10.7	8.8	0.758
Diabetes (%)	18.1	27.7	0.139
Past history of CVD (%)	18.1	10.8	0.185
Systolic blood pressure (mmHg)	138 ± 19	148 ± 18	0.068
Diastolic blood pressure (mmHg)	75 ± 10	76 ± 10	0.891
Serum creatinine (mg/dL)	9.7 ± 3.2	9.4 ± 2.3	0.644
Blood urea nitrogen (mg/dL)	54.0 ± 12.2	57.4 ± 12.3	0.095
Albumin (g/dL)	3.5 ± 0.4	3.6 ± 0.3	0.313
Hemoglobin (g/dL)	9.2 ± 1.1	9.7 ± 0.9	0.069
Total cholesterol (mg/dL)	181 ± 36	174 ± 27	0.232
HDL cholesterol (mg/dL)	42.5 ± 13.0	45.9 ± 15.5	0.251
Triglycerides (mg/dL)	147 ± 76	131 ± 106	0.401
Calcium (mg/dL)	9.2 ± 1.0	9.0 ± 0.8	0.278
Phosphate (mg/dL)	5.1 ± 1.3	5.0 ± 1.3	0.521
Intact PTH (mg/dL)	197 ± 150	146 ± 135	0.051
Use of phosphate binder (%)	92.3	84.1	0.446
Use of vitamin D analogs (%)	61.5	62.2	0.964
Use of ESA (%)	23.5	45.8	0.090

CVD: cardiovascular disease: HDL: high-density lipoprotein: PTH: parathyroid hormone: ESA: erythropoiesis-stimulating agents. Mean ± SD, Median (min-max).

**Table 2:** Hazard ratios for clinical parameters derived by Cox proportional hazard analysis.

	Univariate		Multivariate	
	HR (95% CI)	*P* value	HR (95% CI)	*P* value
Age (per 10 years increase)	1.68 (1.33–2.13)	<0.001	1.88 (1.34–2.64)	<0.001
Past history of CVD (%)	2.42 (1.29–4.53)	0.006	1.75 (0.83–3.68)	0.141
Albumin level (per 1SD increase)	0.62 (0.48–0.81)	<0.001	0.67 (0.49–0.91)	0.011
Triglycerides level (per 1SD increase)	0.60 (0.38–0.95)	0.029	0.73 (0.44–1.22)	0.232
Modality of dialysis (PD)	1.14 (0.65–1.88)	0.720	0.88 (0.44–1.74)	0.710

HR: hazard ratio: CI confidence interval: SD: standard deviation: CVD: cardiovascular disease: PD: peritoneal dialysis.
